# Effects of early adjunctive pharmacotherapy on serum levels of brain injury biomarkers in patients with traumatic brain injury: a systematic review of randomized controlled studies

**DOI:** 10.3389/fphar.2023.1185277

**Published:** 2023-05-05

**Authors:** Noha O. Mansour, Mohamed Hassan Elnaem, Doaa H. Abdelaziz, Muna Barakat, Inderpal Singh Dehele, Mahmoud E. Elrggal, Mahmoud S. Abdallah

**Affiliations:** ^1^ Clinical Pharmacy and Pharmacy Practice Department, Faculty of Pharmacy, Mansoura University, Mansoura, Egypt; ^2^ School of Pharmaceutical Sciences, Universiti Sains Malaysia, Minden, Malaysia; ^3^ School of Pharmacy and Pharmaceutical Sciences, Ulster University, Coleraine, United Kingdom; ^4^ Pharmacy Practice and Clinical Pharmacy Department, Faculty of Pharmacy, Future University in Egypt, Cairo, Egypt; ^5^ Department of Clinical Pharmacy and Therapeutics, Faculty of Pharmacy, Applied Science Private University, Amman, Jordan; ^6^ MEU Research Unit, Middle East University, Amman, Jordan; ^7^ School of Pharmacy, University of Birmingham, Birmingham, United Kingdom; ^8^ College of Pharmacy, Umm Al-Qura University, Makkah, Saudi Arabia; ^9^ Department of Clinical Pharmacy, Faculty of Pharmacy, University of Sadat City, Sadat City, Egypt

**Keywords:** neuron-specific enolase, biomarkers, S100 ß, NSE, GFAP, UCHL1, memantine, metformin

## Abstract

**Objectives:** Traumatic brain injury (TBI) is one of the top causes of morbidity and mortality worldwide. The review aimed to discuss and summarize the current evidence on the effectiveness of adjuvant neuroprotective treatments in terms of their effect on brain injury biomarkers in TBI patients.

**Methods:** To identify relevant studies, four scholarly databases, including PubMed, Cochrane, Scopus, and Google Scholar, were systematically searched using predefined search terms. English-language randomized controlled clinical trials reporting changes in brain injury biomarkers, namely, neuron-specific enolase (NSE), glial fibrillary acid protein (GFAP), ubiquitin carboxyl-terminal esterase L1 (UCHL_1_) and/or S100 beta (S100 ß), were included. The methodological quality of the included studies was assessed using the Cochrane risk-of-bias tool.

**Results:** A total of eleven studies with eight different therapeutic options were investigated; of them, tetracyclines, metformin, and memantine were discovered to be promising choices that could improve neurological outcomes in TBI patients. The most utilized serum biomarkers were NSE and S100 ß followed by GFAP, while none of the included studies quantified UCHL_1_. The heterogeneity in injury severity categories and measurement timing may affect the overall evaluation of the clinical efficacy of potential therapies. Therefore, unified measurement protocols are highly warranted to inform clinical decisions.

**Conclusion:** Few therapeutic options showed promising results as an adjuvant to standard care in patients with TBI. Several considerations for future work must be directed towards standardizing monitoring biomarkers. Investigating the pharmacotherapy effectiveness using a multimodal biomarker panel is needed. Finally, employing stratified randomization in future clinical trials concerning potential confounders, including age, trauma severity levels, and type, is crucial to inform clinical decisions.

**Clinical Trial Registration:** [https://www.crd.york.ac.uk/prospero/dis], identifier [CRD42022316327].

## 1 Introduction

Traumatic brain injury (TBI) is a leading cause of morbidity and mortality globally (Dewan et al., 2018). Annually, an estimated 50–60 million new cases are reported. Survivors typically suffer from post-traumatic challenges ranging from neurological and psychosocial issues to permanent disabilities (Quaglio et al., 2017). Neural damage post-brain trauma falls into two categories: primary insult, which is directly caused by mechanical forces in the initial injury and delayed secondary insult, which results from a subsequent cascade of cellular and biochemical events (Ng and Lee, 2019). The primary pathophysiology of secondary injury is still lacking; however, mitochondrial dysfunction and apoptotic cell death are mechanistically assumed to be the primary contributors (Ray et al., 2002). Oxidative stress (Khatri et al., 2018) and neuroinflammation (Simon et al., 2017) also play an important role in this process. Brain edema and the resulting elevated intracranial pressure are major contributors to the adverse prognosis in patients with brain trauma. Therefore, early preventive strategies against secondary brain injury are crucial for improving the clinical outcomes of those patients (Jha et al., 2019).

Current management relies on immediate interventions to stabilize patients, such as decompressive craniectomy (Hawryluk et al., 2020), nutrition management (Vella et al., 2017), or prophylactic hypothermia (Chen et al., 2019), but their benefits did not presume to reduce the secondary neural damage. So far, no pharmacotherapy could modulate the primary insult. Nevertheless, therapeutic interventions in the acute phase have focused on restricting the cellular cascade of secondary insults. Numerous clinical studies have searched for early adjunctive treatments to prevent further neuronal damage and improve functional recovery (Langham et al., 2003; Lu et al., 2012; Zeng et al., 2015; Synnot et al., 2018). The clinical trials failure has been linked to the absence of central biomarkers for medication monitoring, TBI heterogeneity, and the limited translatability of TBI preclinical studies (Wang et al., 2018).

The pathophysiologic processes of TBI involve axonal injury, neuronal cell body injury, and microglia responses. To monitor these different processes, thus far, a panel of biomarkers has been recognized. Neuron-specific enolase (NSE) is a neuronal acute injury biomarker. It is one of the most clinically used biomarkers to monitor the effectiveness of therapeutic interventions (Yokobori et al., 2013). Enolase is a key enzyme of glycolysis and gluconeogenesis, two vital metabolic pathways for cellular functions. Neuron-specific enolase is expressed abundantly in neuronal cell bodies. In intact neurons, NSE is not normally secreted in extracellular fluids; however, when neurons are damaged, NSE is upregulated to maintain homeostasis. Higher NSE levels can be detected in the serum in patients with neuronal injury. This extracellular release is caused partly by leakage from injured neurons. The upregulation of NSE also contributes to this elevation to initiate repair mechanisms. Therefore, NSE directly assesses functional damage to neurons (Cheng et al., 2014). The lack of brain specificity is a major limitation of NSE. Neuron-specific enolase is abundant in RBCs, which may produce false positive results. (Graham et al., 2016). Higher NSE concentrations (>20 μg/L) were found to be associated with higher mortality rates in patients with moderate and severe brain injuries (Cheng et al., 2014).

S-100β protein, the β subunit of a calcium-binding protein produced by astrocytes, is one of the most well-characterized biomarkers of TBI. Increased S-100β serum levels after brain injury were linked to glial damage. S-100β protein peaks early in the first 6 h, and a second peak, more sensitive to neuronal injury severity, occurs after 48 h post-injury (Slavoaca et al., 2020). High serum levels of S-100β had significant correlation with injury severity and prognosis; levels ranging from 1.38 μg/L to 10.5 μg/L and from 2.16 μg/L to 14.0 μg/L were connected with 100% specificity for mortality and a Glasgow outcome score (GOS) ≤3 respectively (Mercier et al., 2013).

Glial fibrillary acid protein (GFAP) is a key intermediate filament-III protein uniquely found in astrocytes, one of the glial cells. It strengthens the cytoskeleton structure for glial cells and supports the integrity of the blood-brain barrier (Abdelhak et al., 2022). The primary strength of GFAP as a brain injury biomarker is brain specificity; it is only found within the CNS (Graham et al., 2016). Like S-100β protein, high serum levels of GFAP are quickly detected in the first 24 h post-injury (Slavoaca et al., 2020).

Ubiquitin C-terminal hydrolase (UCHL1) is a cysteine protease predominately expressed in neuronal cell bodies. Due to its specific expression in brain tissue, high serum levels of UCHL1 have been used as a marker of neuronal cell body injury.

Several neuroprotective agents have been evaluated regarding their modulating effects on brain injury biomarkers. Yet, the evidence remains inconclusive. Recent reviews of potential neuroprotective candidates relied on evaluating their effects on the widely used GOS and/or its extended version, GOS-E (Begemann et al., 2020; Liu M. et al., 2020; Solla and Paiva, 2021). There have been many criticisms of their subjectivity in measuring recovery from TBI (Shukla et al., 2011). These scales rely on self-assessment or assessment of a caregiver rather than quantifiable measurements of disability (Iyer et al., 2009). The quantitative outcome measures would be beneficial, although these may be more expensive and time-consuming to implement(Ma et al., 2016). Other reports evaluated the survival benefits and the incidence of unfavorable neurological outcomes. Despite their importance, these measures could be confounded by factors such as injury severity level (Okidi et al., 2020) and age (Hukkelhoven et al., 2003). Based on the clinical utility of brain injury biomarkers and their ability to inform therapeutic decision-making in patients with central trauma, this review aimed to comprehensively discuss and summarize the available evidence from randomized controlled clinical trials (RCTs) that evaluated novel options in terms of their effects on acute injury biomarkers to bridge the knowledge gap and allow new therapy development.

## 2 Materials and methods

This systematic review’s findings were reported using the Preferred Reporting Items for Systematic Reviews and Meta-Analyses (PRISMA) guideline.

### 2.1 Data sources and search strategy

**FIGURE 1:**
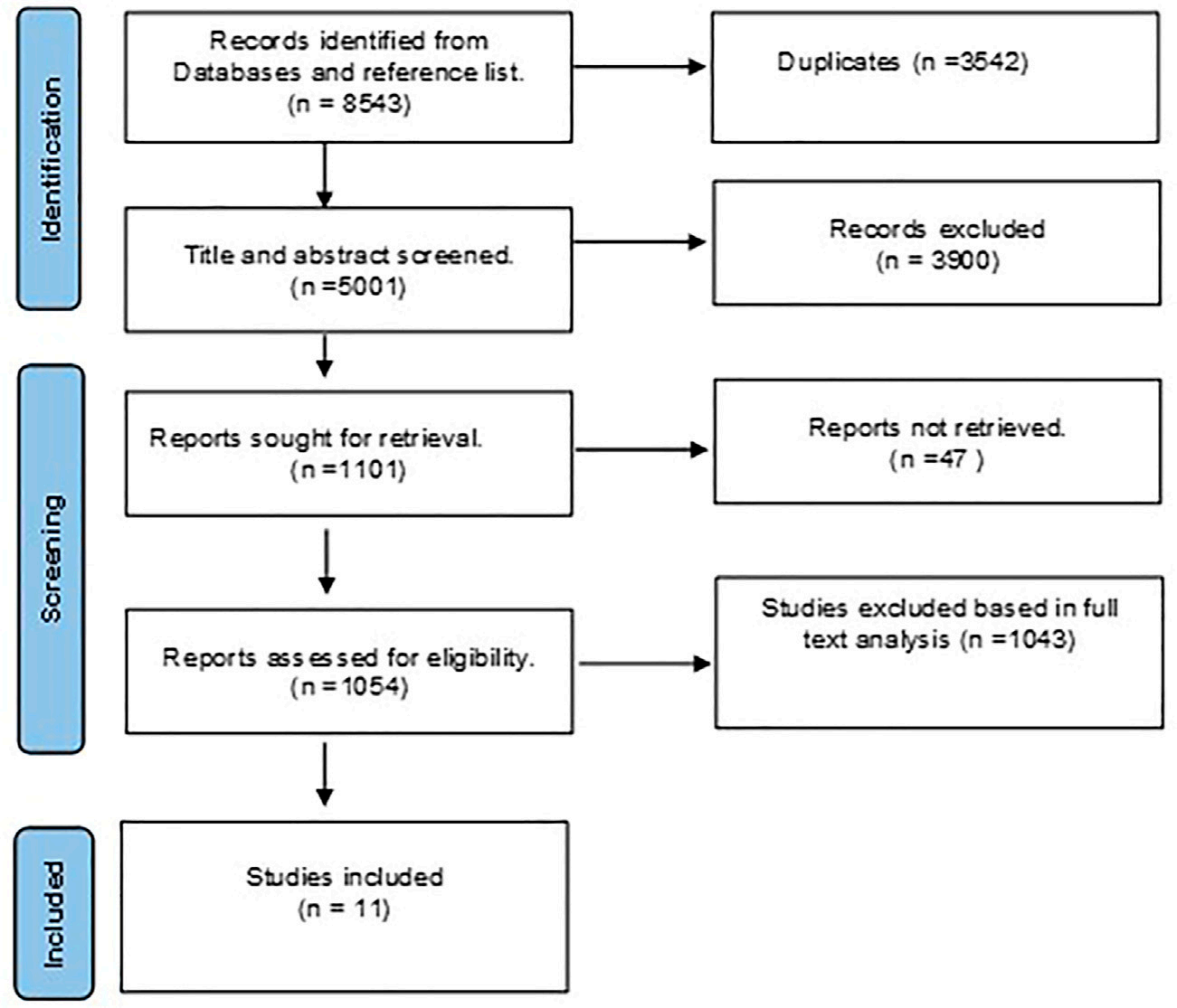
Preferred reporting items for systematic reviews and metanalysis (PRISMA) flowchart for eligible studies selection process.

The study protocol was registered in the international prospective register of systematic reviews PROSPERO registry (registration number: CRD42022316327). A systematic search was conducted on four major electronic databases: PubMed, Cochrane, Scopus, and Google Scholar. Clinical trials published till March 2022 were included (Figure 1). The following terms were searched: “head trauma”, “traumatic brain injury”, “brain trauma”, “neuron-specific enolase”, NSE, “Glial fibrillary acidic protein”, GFAP, “S100β", UCHL, “Ubiquitin C-terminal hydrolase”, and “brain injury biomarkers”. To retrieve relevant trials, registries such as ClinicalTrials.gov were searched for trials undertaken in TBI patients. The search was limited to trials investigating the additive effects of any pharmacotherapy on potentially modulating injury biomarkers in those patients. To increase the likelihood of finding additional relevant papers, the reference lists of the retrieved articles as well as the “related articles” feature in PubMed, were reviewed. A manual search of reference lists for all related reviews was also conducted. Simvastatin, cilostazol, N-acetyl cysteine, and melatonin were searched manually based on their previously reported neuroprotective properties in TBI (Begemann et al., 2020).

### 2.2 Study screening and selection

Original studies published on pharmacotherapeutic options in TBI patients were eligible for inclusion. For inclusion eligibility, the records were separately examined by two authors (MHE and NOM). A third author (DHA) was brought in to settle any disputes, and all decisions were reached by consensus. Eligible studies were obtained in full text to be included, and their methodological quality were evaluated.

### 2.3 Data extraction

Data from the selected studies were extracted using a standardized form. The following basic information was extracted from the RCTs: name of the lead author, year of publication, site, study design (randomization, blinding), sample size, patient population (age; severity of injury according to GCS scores), interventions (type of pharmacological agent; dosing regimen), outcomes (monitored biomarkers, time of sampling), main results, and conclusions.

### 2.4 Eligibility criteria

Studies and trials meeting the following criteria were included:

• *Study design*: RCTs published in the full-text version.
• *Population*: patients with a clinical diagnosis of TBI or diffuse axonal injury (DAI) regardless of severity.
• *Interventions*: pharmacological agent with neuroprotective effects (such as delayed neuroinflammation, neuronal cell death, and neurological Dysfunction). Agents could be administered in any regimen initiated immediately in the acute phase of TBI.
• *Comparators:* active comparator, placebo or no drugs
• *Outcomes*: The change in an acute brain injury biomarker.

The exclusion criteria were as follows:

•  Studies evaluated non-pharmacological drug therapy such as decompressive craniectomy, therapeutic hypothermia, and nutritional supplements.
•  Assessing the efficacy of adjuvant treatment based on functional and/or clinical outcomes such as GOS, GOS-E, and mortality.
•  Studies in polytrauma patients or the presence of accompanying other neurodegenerative diseases.
•  Studies of interventions implemented in the post-acute and chronic phases.

### 2.5 Quality assessment

Each RCT’s level of evidence was assessed using the Cochrane Risk-of-Bias (RoB) tool to identify any potential bias risks (Higgins and Altman, 2008). Two authors independently assessed the included studies’ methodological quality using this approach. A third author was consulted in case of disagreement concerning the risk of bias. The following items were evaluated:

#### 2.5.1 *Generation of random sequences*:

Methods used to generate the sequence.

#### 2.5.2 Allocation concealment:

Assessment of whether intervention allocations could have been foreseen before or during enrollment. Accurate reporting of the technique utilized to mask the allocation sequence was evaluated.

#### 2.5.3 *Blinding*:

Description of procedures taken to blind trial patients and investigators from knowledge of allocated study intervention.

#### 2.5.4 *Blinding of outcome assessment*:

Description of procedures to mask outcome assessors from knowledge of allocated study intervention

#### 2.5.5 *Incomplete outcome data*:

Describe the accuracy of the outcome information for each primary outcome, considering attrition and analytical exclusions.

#### 2.5.6 Selective reporting:

*Assessment* of the possibility of selective outcome reporting by the authors.

Each of the aforementioned items was assigned a low, high, or unclear risk level. To assess selection bias, randomization sequence generation and allocation concealment were used. Blinding participants and/or investigators represented the risk of performance bias. Blinding outcome assessors represented the risk of detection bias, and each trial’s reporting bias was assessed using incomplete outcome data.

## 3 Results

A total of 8543 studies were discovered during the primary scholarly search. We eliminated 3542 duplicate studies using EndNote. The remaining studies were reviewed, and 1054 qualified for full-text analysis. As a result, 1043 studies were eliminated because they failed to meet the inclusion criteria. Finally, eleven studies that evaluated different pharmacotherapeutic agents on the prespecified biomarkers in TBI patients were incorporated into this review (Figure 1).

### 3.1 Overview of the included studies

Table 1 illustrates an overview of the summary of the studies based on trial design, study population, and conclusions. Erythropoietin, progesterone, and tetracyclines (two studies each); N-acetyl cysteine, fluid therapy; metformin, L-carnitine, and memantine (one each). All the included studies were single-center studies distributed as follows: Iran (*n* = 4), unspecified (*n* = 2), Egypt (*n* = 1), Canada (*n* = 1), China (*n* = 1), Indonesia (*n* = 1), and United States (*n* = 1). The review included eleven RCTs. Most studies included small sample sizes ranging from 14 to 159 patients. Regarding injury severity, the GCS was used to classify TBI subjects as severe (GCS ≤8), moderate (GCS 9–12), and mild (GCS 13–15) in all reports (Wang et al., 2018). Seven RCTs enrolled patients in one category of TBI severity (6 RCTs in patients with severe injury (Baker et al., 2009; Li et al., 2016; Clark et al., 2017; Mahmoodpoor et al., 2018; Taheri et al., 2019; Mahyudanil et al., 2020); and 1 RCT in patients with moderate injury (Mokhtari et al., 2018)). Four articles reported data that applied to multiple severity categories (Nirula et al., 2010; Shahrokhi et al., 2016; Koulaeinejad et al., 2019; Mansour et al., 2021).

**TABLE 1:** Summary of the included studies.

Trial		Patient population	Intervention	Comp.	Outcome(s)	Conclusion
1	Mansour et al. (2021) Egypt	(*n* = 50) TBI patients, GCS (3–12)	100 mg doxycycline BID for 7 days	Placebo	Difference between tde two study groups in mean NSE serum levels at day 7.	NSE serum levels in tde doxycycline group were significantly lower tdan in tde control group.
2	Mahyudanil et al. (2020) Indonesia	(*n* = 40) TBI patients, GCS of 4–8	Single dose progesterone 1 mg/kg	Placebo	Difference in serum level of S-100β in 24 h and 96 h between the two arms of the study.	Insignificant change in serum level of S-100β was observed.
3	Koulaeinejad et al. (2019) Iran	(*n* = 40) TBI patients, GCS ≤12.	100 mg minocycline BID for 7 days	Placebo	Changes in level of NSE and S100 β from day 1 to day 5 after randomization.	The reduction in serum NSE and S100 β levels from baseline to day 5 was statistically significant in the minocycline group but not in the placebo group.
4	(Taheri et al., 2019) Iran	(*n* = 30) TBI patients, GCS ≤8	1 g metformin/12 h for 5 days.	No drug	5-day post-trauma serum concentration profile (24 h, 48 h, 72 h and 120 h) of S100B and GFAP.	Significantly lower S100b in patients allocated to metformin. GFAP values did not differ between groups at all study time points
5	Mahmoodpoor et al. (2018)	(*n* = 58) TBI patients, GCS ≤8.	2 g L-carnitine once daily for 7 days	Placebo	Difference between the study groups in mean NSE serum levels at day 3.	L-carnitine failed to reduce serum NSE levels in patients with TBI.
6	Mokhtari et al. (2018) Iran	(*n* = 68) TBI patient, GCS (9–12)	30 mg memantine BID for 7 days	No drug	Difference in NSE serum levels at days 1, 3, and 7 post randomizations.	Memantine significantly reduced NSE levels by day in patients with moderate TBI.
7	Clark et al. (2017) United States	(*n* = 14) TBI pediatric patients, GCS ≤8	Combined N-acetylcysteine and probenecid	Placebo	Difference in serum levels of NSE and GFAP, in 24 h and 96 h between the two arms of the study.	Brain injury biomarkers were deemed comparable between the two groups (*p* = 0.441).
8	Shahrokhi et al. (2016) Iran	(*n* = 32) DAI patients, GCS ≤12.	1 mg/kg IM BID progesterone for 5 days.	Placebo	Difference between the two study groups in mean NSE serum levels at days 1 and 6.	Progesterone did not change the serum level of NSE between the study groups.
9	Li et al. (2016) China	(n = 159) TBI patients, GCS ≤8.	100 U/kg erythropoietin SC for 12 days	Normal Saline	The difference between groups in S100B and NSE levels	Serum NSE and S-100ß protein levels were lower in patients who received erythropoietin.
10	(Nirula et al., 2010) United States	(*n* = 16) TBI patients, GCS ≤13.	Erythropoietin (40,000 Units IV) within 6 h of injury.	placebo	Difference between groups in serum concentrations of S100B and NSE at day 1, 2, 3,4 and 5 post resuscitation	Erythropoietin did not impact NSE (*p* = .89) or S100 B (*p* = 0.53) levels compared to the placebo.
11	Baker et al. (2009) Canada	(*n* = 64) TBI patients, GCS ≤8.	250 mL 7.5% hypertonic saline +6% dextran70 (HSD)	0.9% normal saline (NS)	Difference between groups in serum concentrations of S100B and NSE at 12, 24, and 48 h post-resuscitation.	Compared with NS resuscitation, S100B and NSE were two and threefold lower in HSD-treated patients and normalized within 12 h.

### 3.2 Biomarkers of TBI

All included studies measured biomarkers in the serum of the patients. The most commonly used biomarker (n = 9) was NSE (Baker et al., 2009; Nirula et al., 2010; Li et al., 2016; Shahrokhi et al., 2016; Clark et al., 2017; Mahmoodpoor et al., 2018; Mokhtari et al., 2018; Koulaeinejad et al., 2019; Mansour et al., 2021), followed by S100 ß (n = 6) (Baker et al., 2009; Nirula et al., 2010; Li et al., 2016; Koulaeinejad et al., 2019; Taheri et al., 2019; Mahyudanil et al., 2020). Glial fibrillary acid protein was monitored in two studies (Clark et al., 2017; Taheri et al., 2019). None of the included studies quantified UCHL1. Five of the included RCTs reported change over time only in one acute injury biomarker, six studies reported changes in two biomarkers, and none of the included articles monitored more than biomarkers. Commercially available enzyme-linked immunoassay analysis kits assessed serum biomarkers in ten studies, while monoclonal immunoluminometric assay was used only once. Studies greatly varied in terms of the number and timing of follow-up measurements. Repeated longitudinal assay of biomarkers levels was detected in all included studies. The time frame of the assay greatly varied between studies. The assay timing started on day one (randomization day) and was followed thereafter by serial measurements for up to 3 months. Detailed description of biomarker measurement in each study is depicted in Table 1.

### 3.3 Methodological quality of studies

**FIGURE 2:**
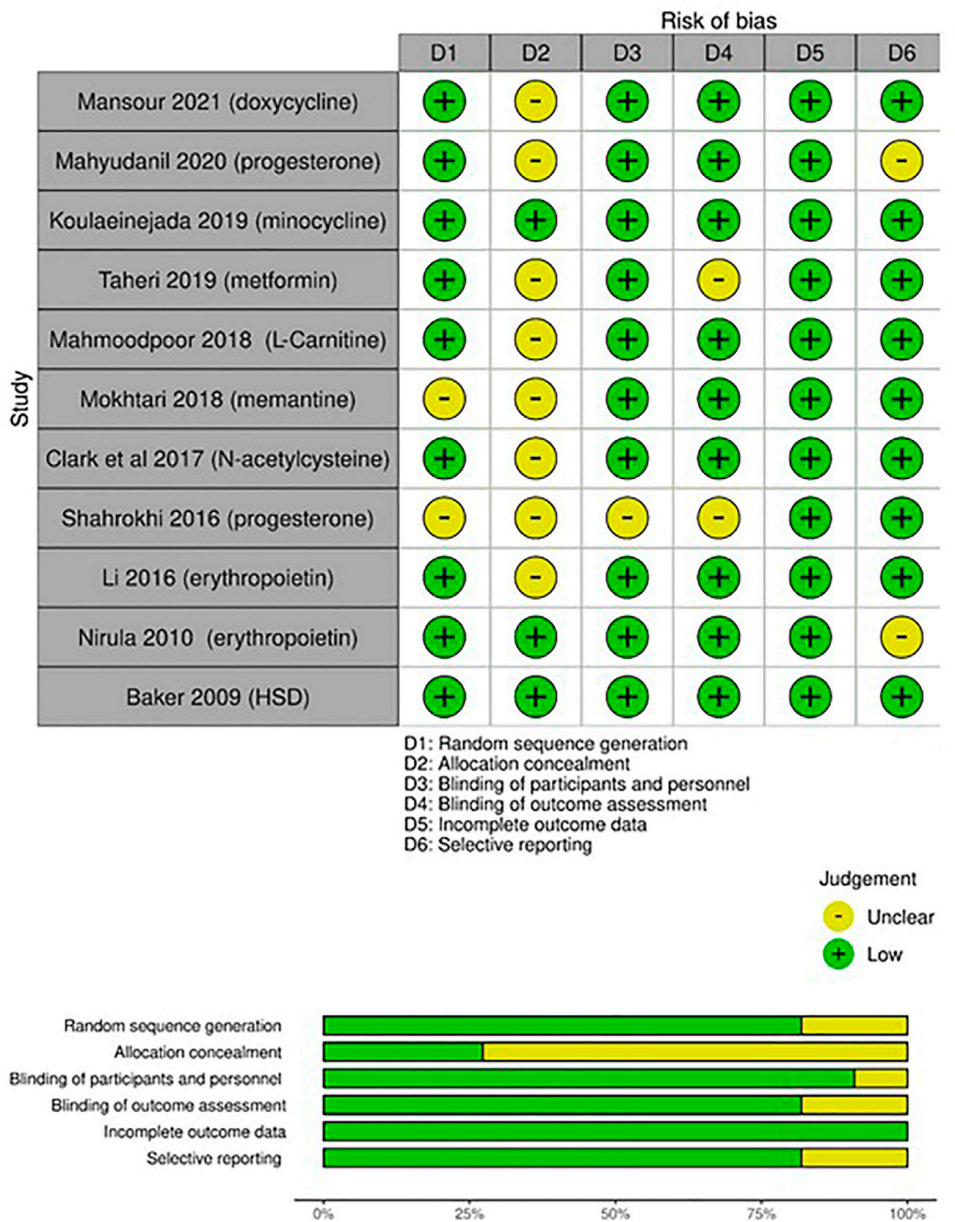
Risk of bias assessment (RoB) according to the Cochrane RoB tool for randomized controlled trials (RCTs): risk of bias per item for each study; risk of bias per item presented as percentages across all included RCTs.

The Cochrane RoB assessment tool was used to evaluate the quality of the RCTs. The quality of included RCTs and the RoB per item are presented in Figure 2. Methods used for random sequence generation were detailed in more than 80% of the included studies. Nonetheless, there was a lack of adequate description of the methods used to conceal the allocation sequence in about 75% of the included studies. Thus, a high risk of selection bias may be presumed. Differences in the care received by the intervention and control groups were minimized in most of the included studies by properly describing the masking procedures. Low risk of detection bias was noted among the included RCTs. Most of the included studies had risks of undermining the validity mainly due to small sample sizes and lack of description of methods of estimating sample size and power.

## 4 Discussion

This research investigated whether brain injury biomarkers may be normalized in TBI patients by using drugs that have neuroprotective properties. S100B and NSE were the biomarkers that were mostly monitored, followed by GFAP. Similar findings have been reported by Marzano et al., who summarized the recent findings in the medical literature about the diagnostic and prognostic value of brain injury biomarkers in the pediatric population with TBI (Marzano et al., 2022). Eleven studies were summarized for qualitative analysis to understand better how current literature supports the effectiveness of early adjunctive pharmacotherapy, including tetracyclines, progesterone, erythropoietin, metformin, L-carnitine, resuscitation fluid, and memantine.

### 4.1 Tetracyclines

The merits of tetracyclines as neuroprotective agents have been previously recognized in a variety of neurodegenerative conditions, including Parkinson’s disease (Santa-Cecília et al., 2019), Alzheimer’s disease (Balducci et al., 2018), and multiple sclerosis (Minagar et al., 2008). Our findings included two studies that examined the short-term effectiveness of additional tetracyclines in TBI patients: doxycycline and minocycline. Both agents were used in regular, approved doses (100 mg BID). Consistent results and promising effects in terms of the impact on the brain injury biomarkers have been reported in both RCTs. According to Mansour et al., NSE levels were significantly lowered in patients assigned to the doxycycline group compared to those assigned to the placebo (12.81 ng/mL versus 16.43 ng/mL (*p* = 0.003). Additionally, significant larger proportion of patients had normalized NSE levels with early doxycycline administration (≤12 ng/mL) (45% in doxycycline group vs 25% in Placebo) (Mansour et al., 2021). Likewise, Koulaeinejad et al. noted a marked reduction in NSE from baseline to day 5 with early minocycline administration (*p* = 0.01) (Koulaeinejad et al., 2019). The exact molecular mechanism behind the neuroprotective effects of tetracyclines is still unclear. However, it is reasonable to suggest that inhibition of apoptosis (Elewa et al., 2006), repair of the blood-brain barrier (Plane et al., 2010; Malek et al., 2020), and the neuro-anti-inflammatory actions (Elewa et al., 2006) (Chaves Filho et al., 2021) are thought to be the main contributing factors to the reported benefit. These findings provide a sufficient basis for further investigations. Nevertheless, both studies were limited by the small sample size and the pilot study design. Randomizing patients with moderate and severe brain trauma limits data generalizability to those with mild TBI. Larger clinical trials, with stratified randomization according to severity, are crucial to informing the drug development process better. In addition, the promises shown in the acute phase of TBI encourage prolonged administration to modulate functional recovery and mortality.

### 4.2 Erythropoietin

Erythropoietin, a multi-functional cytokine released in the kidney and CNS, has been proposed as a potential neuroprotective agent (Schober et al., 2018). Erythropoietin has been shown to be effective against several early mediators of secondary brain injury in preclinical studies, mainly via reducing pro-inflammatory cytokines and enhancing the anti-inflammatory cytokines in brain tissue (Zhou et al., 2017; Silva et al., 2021). Besides, preserving brain oxygenation by erythropoietin is involved in its anti-oxidative and glial protective actions in patients with TBI (Lee et al., 2019). These aforementioned properties might contribute to the erythropoietin-induced neuroprotective effects. Supporting the previous preclinical data, our results included one RCT (*n* = 159) with a positive outcome. In this study, Li et al. reported the effectiveness of low-dose erythropoietin in patients with severe TBI. Lower levels of NSE and S100ß were detected in patients who were randomized to receive erythropoietin (administered in five doses (100 units/kg) for 12 days) compared to the control (Li et al., 2016).

Conversely, another earlier study pilot study (*n* = 16) by Nirula et al. (Nirula et al., 2010) failed to demonstrate similar efficacy with erythropoietin use (40,000 Units IV within 6 h) on the same biomarkers. The discrepancy in the reported outcomes across the two RCTs included in the present review could be explained by the differences in sample size, dosing regimen, and severity of neuronal injury among recruited patients. Two meta-analyses recently concluded that early use of erythropoietin lowered the mortality risk (Lee et al., 2019; Liu C. et al., 2020). Nonetheless, no difference was found concerning the enhancement of neurological outcomes. The dose and timing of erythropoietin injections varied considerably across the reviewed studies in the previous reviews. Given the cost, more research is required to define the merits of erythropoietin use in this particular clinical indication.

### 4.3 Progesterone

Steroid hormones are produced mainly by adrenal glands and gonads and control the function of several target organs, including the brain. In addition, some steroids are *de novo* synthesized glial cells called “neuro-steroids. Circulating progesterone passes the blood-brain barrier owing to its lipophilic properties. It is also a neurosteroid locally synthesized by the glial cells in the brain tissue. Growing evidence from reviews of experimental research in animal models indicates that progesterone has a neuroprotectant effect. These benefits might be explained by reducing brain edema (Wang et al., 2013), blood-brain barrier stabilizing effect (Si et al., 2014), and reduction of the inflammatory response post-trauma (Chen et al., 2008; Lei et al., 2014). However, evidence about the effects of progesterone on brain injury biomarkers is scarce. Only two small-scale studies examined the impact of progesterone on NSE and reported negative results. Due to the small sample size, these findings should be interpreted cautiously. According to a Cochrane review of three RCTs, the neurologic prognosis of TBI patients may be enhanced with progesterone. Nevertheless, this evidence is still insufficient, and further multicenter trials are required (Ma et al., 2016)

### 4.4 Metformin

Microglia, the CNS macrophages, are involved in neurodegenerative disease pathogenesis. Microglial cells become activated within minutes of brain injury. Once activated, microglia secrets pro-inflammatory cytokines such as interleukins, TNF-α, and free radicals (Ramlackhansingh et al., 2011). Suppressing the pro-inflammatory microglial cells has been recently targeted to attenuate the rate of inflammation and consequent neurological deficit in animal models (DiBona et al., 2021; Bourget et al., 2022). Apart from suppressing microglial activation, a growing body of evidence proves that metformin neuroprotective effects are attributable to AMP-activated protein kinase activation (Rahimi et al., 2020; Zhang et al., 2022; Zhou et al., 2022). The efficacy of metformin therapy was investigated in one RCT. Administration of 1 g metformin BID for 5 days. Analysis of the S100ß level revealed statistically significant decreases in values toward normal levels in the intervention group. Contrarily, the dynamics of serum GFAP levels in the two study groups were not statistically different at all study time points. Safety data reported that metformin is tolerable, with no events of hypoglycemia or lactic acidosis reported in study participants. Considering the possible disease-modifying effects on the pharmacokinetics of metformin in patients with severe TBI. Taheri et al. showed that the intervention group needed a longer time to reach its maximum metformin concentration than healthy subjects (Taheri et al., 2019). Thus, the time required to reach the site of action in the CNS may be prolonged. Future larger studies of metformin use with prokinetic agents might augment the shown benefit.

### 4.5 Memantine

The N-methyl-D-aspartate (NMDA) glutamate receptors are linked to neuronal cell death through different mechanisms, including excitotoxicity and apoptosis. Activation of glutamate receptors increases calcium influx, resulting in neuronal apoptosis. On the other hand, significant glutamate release results in magnesium loss in the glutamate receptor’s ion channel. Consequently, neuronal cells depolarize, swell, and necrotize (Khan et al., 2021). Preclinical models indicate that glutamate-mediated excitotoxicity is pivotal in the secondary injury cascade. Mokhtari et al. investigated the effect of NMDA receptor blocking *via* memantine (30 mg BID for 7 days) in moderate TBI patients (Mokhtari et al., 2018). A promising neuroprotective effect was reported with early memantine use (within the first 24 h post-injury). Serum NSE levels in the memantine group were significantly lower than in the control group from day 0 to day seven (*p* = 0.009). This effect was linked to a significant daily improvement in the patient’s GCS scores.

### 4.6 N acetylcysteine

N-acetyl l-cysteine, a sulfur-containing amino acid, replenishes glutathione and may lessen subsequent brain damage. Reliable preclinical data showed a strong association between N-acetyl cysteine administration and improved neurological outcomes. Preventing sequelae from induced TBI was mainly illustrated by counteracting the increased oxidative stress, promoting redox-controlled cell signaling, and reducing immuno-inflammatory reactions post-trauma (Bhatti et al., 2018). The effectiveness of N-acetyl cysteine has been evaluated in different neurodegenerative diseases (Tardiolo et al., 2018). Despite n-acetyl cysteine’s low blood-brain barrier permeability, its actions as neuroprotectants when combined with probenecid have been investigated in a pediatric placebo-controlled trial (Clark et al., 2017). The serum levels of neuro-injury biomarkers in the participants did not differ after administration of n-acetyl cysteine, compared with a control group. Given the dearth of studies, conclusive results could not be elucidated. Further research is needed. Its amide derivative, N-acetylcysteine amide, has increased BBB permeability, implying increased CNS bioavailability (Matthiesen et al., 2021). However, it has not been clinically investigated in brain trauma patients.

### 4.7 The evidence gap and future research implications

Despite the availability of several pharmacotherapeutic options, TBI management is still challenging, and many areas of uncertainty persist. The section highlights four main selected issues in the current studies. Also, recommendations that help address these issues in upcoming clinical studies are underlined to guide the development of evidence-based recommendations. Table 2 presents a summary of these pitfalls and the relevant recommendations to be considered in future research.

**TABLE 2:** Common pitfalls in TBI studies and recommendations for future research.

Gaps in evidence/pitfalls	Recommendations
The use of a single biomarker is not sufficient for monitoring patients over time across the TBI spectrum	Biomarker panels are needed for better assessment of diverse clinical phenotypes of patients with TBI. Including markers from different modalities is needed for effective joint benefit.
Heterogeneity in the monitoring of brain injury biomarkers	• More research is needed to explore the optimal sampling protocol for each biomarker.
	• A protocolized algorithm that guides sampling based on the kinetics of each biomarker.
Confounding Factors	Variations could be minimized via employing stratified randomization based on possible confounders such as: • Type and severity of the injury.• Renal functions.
Lack of data from RCTs about the effect of adjuvant pharmacotherapies on different brain injury biomarkers	Exploring potential benefits of the promising agents reported in the current review, such as metformin and tetracyclines, on other emerging biomarkers.
Clinical investigation of novel therapeutic approaches	Promising agents for future clinical research • High dose vitamin D • Melatonin. • Nicotinamides.

#### 4.7.1 Heterogeneity in the monitoring of brain injury biomarkers

The timing of blood samples for biomarkers is unlikely to be crucial in some neurodegenerative diseases (Zetterberg and Bendlin, 2021). On the contrary, sampling timing is critical in TBI (Bogoslovsky et al., 2016; Thelin et al., 2017). So far, there are only a few kinetic studies of blood biomarkers after TBI. Moreover, trials with primary endpoints rely on monitoring response to therapy based on biomarkers levels require a protocolized algorithm that directs sampling based on each biomarker’s kinetics. This would lessen the effect of variances on the reported outcome.

#### 4.7.2 Confounding factors

Type and severity of injury: In mild TBI, patients might have no or minimal disruption of the blood-brain barrier, while it occurs with moderate or severe brain injury in about 40% of the cases (Saw et al., 2014; Hier et al., 2021). This conceivably affects levels of biomarkers that enter the peripheral blood. Further research should carefully consider the severity of injury to enhance the external validity of their findings.

Renal functions: some biomarkers are renally eliminated, and kidney dysfunction can prolong the elimination half-life in the blood and elevate biomarker blood levels (Hier et al., 2021).

#### 4.7.3 Lack of data from RCTs about the effect of adjuvant pharmacotherapies on different brain injury biomarkers

Exploring the potential benefits of the promising agents reported in the current review, such as metformin and tetracyclines, on other emerging novel biomarkers, neurofilament light chain protein (NF-L) (Halbgebauer et al., 2022), and Ubiquitin C-terminal hydrolase-L1 (UCH-L1) (Wang et al., 2021).

#### 4.7.4 Clinical investigation of novel therapeutic approaches

There are several promising agents for future clinical research, as follows:

• High-dose vitamin D offered neuroprotective effects in patients with moderate ischemic stroke (Hesami et al., 2022). Similar action might be expected in patients with TBI.
• Melatonin has anti-apoptotic, brain edema-reducing, and anti-inflammatory effects (Seifman et al., 2014; Lorente, 2017).
• Nicotinamides’ early administration reduced apoptosis and prevented blood-brain barrier damage, yet potential benefits are not clinically examined (Jacquens et al., 2022).

This review provides the latest and comprehensive update for all pharmacotherapeutic choices for early adjuvant therapy in patients with traumatic brain injury. We reported the impact of these therapies on commonly reported brain injury biomarkers. We have also highlighted common issues encountered in the evidence synthesis process and the relevant recommendations for future work in the same area.

Nevertheless, this work has several limitations. First, it did not address delayed axonal injury demyelination markers biomarkers such as neurofilament light chain (NF-L) and myelin basic protein (MBP). The small number of the included studies represents another notable limitation. The heterogeneity in injury severity categories, and measurement timing may affect the overall evaluation of the clinical efficacy of potential therapies.

## 5 Conclusion

This review integrates for the first-time comprehensive evidence on the impact of early adjuvant neuroprotective pharmacological interventions on serum levels of brain injury biomarkers inpatients with brain trauma. The use of multi-modal approach to explore combining different biomarkers is needed in future clinical trials. A unified measurement protocol is highly warranted to inform clinical decisions. Employing stratified randomization concerning potential confounderssuch as age, trauma severity levels, and type are crucial in future investigations.

## Data Availability

The original contributions presented in the study are included in the article/Supplementary Material, further inquiries can be directed to the corresponding author.
